# Prognostic factors in breast phyllodes tumors: a nomogram based on a retrospective cohort study of 404 patients

**DOI:** 10.1002/cam4.1327

**Published:** 2018-02-26

**Authors:** Zhi‐Rui Zhou, Chen‐Chen Wang, Xiang‐Jie Sun, Zhao‐Zhi Yang, Xing‐Xing Chen, Zhi‐Ming Shao, Xiao‐Li Yu, Xiao‐Mao Guo

**Affiliations:** ^1^ Department of Radiation Oncology Fudan University Shanghai Cancer Center Shanghai 200032 China; ^2^ Department of Oncology Shanghai Medical College Fudan University Shanghai 200032 China; ^3^ Department of Pathology Fudan University Shanghai Cancer Center Shanghai 200032 China; ^4^ Department of Breast Surgery Fudan University Shanghai Cancer Center Shanghai 200032 China

**Keywords:** Adjuvant radiotherapy, clinicopathologic features, local recurrence, phyllodes tumor of the breast, surgical treatment

## Abstract

The aim of this study was to explore the independent prognostic factors related to postoperative recurrence‐free survival (RFS) in patients with breast phyllodes tumors (PTBs). A retrospective analysis was conducted in Fudan University Shanghai Cancer Center. According to histological type, patients with benign PTBs were classified as a low‐risk group, while borderline and malignant PTBs were classified as a high‐risk group. The Cox regression model was adopted to identify factors affecting postoperative RFS in the two groups, and a nomogram was generated to predict recurrence‐free survival at 1, 3, and 5 years. Among the 404 patients, 168 (41.6%) patients had benign PTB, 184 (45.5%) had borderline PTB, and 52 (12.9%) had malignant PTB. Fifty‐five patients experienced postoperative local recurrence, including six benign cases, 26 borderline cases, and 22 malignant cases; the three histological types of PTB had local recurrence rates of 3.6%, 14.1%, and 42.3%, respectively. Stromal cell atypia was an independent prognostic factor for RFS in the low‐risk group, while the surgical approach and tumor border were independent prognostic factors for RFS in the high‐risk group, and patients receiving simple excision with an infiltrative tumor border had a higher recurrence rate. A nomogram developed based on clinicopathologic features and surgical approaches could predict recurrence‐free survival at 1, 3, and 5 years. For high‐risk patients, this predictive nomogram based on tumor border, tumor residue, mitotic activity, degree of stromal cell hyperplasia, and atypia can be applied for patient counseling and clinical management. The efficacy of adjuvant radiotherapy remains uncertain.

## Introduction

Phyllodes tumor of the breast (PTB) is a rare type of breast tumor comprising fibrous connective tissue and epithelial tissue. In 2003, the World Health Organization (WHO) recommended naming PTB as phyllodes tumor and divided it into benign, borderline, and malignant types according to five histopathological features: mitotic figures, stromal cell atypia, nature of tumor borders, stromal cell hypercellularity, and overgrowth 1, 2. Specifically, (1) for benign PTB, the tumor showed expansive growth, with a clear tumor border; stromal cells showed obvious hyperplasia, with a sparse arrangement; the cells showed no or mild atypia; mitotic activity was 0–4 mitotic figures/10 high power field (HPF); and bleeding or necrosis was absent; (2) for borderline PTB, the tumor showed expansive or partial infiltrative growth; stromal cells showed overgrowth, with moderate atypia; mitotic activity was 5–9 mitotic figures/10 HPF; and bleeding or necrosis appeared in small areas; and (3) for malignant PTB, the tumor often showed infiltrative growth, with an unclear tumor border, infiltrating the surrounding tissue; stromal cells showed significant overgrowth and obvious atypia, which might be accompanied by heterologous differentiation; mitotic activity was ≥10 mitotic figures/10 HPF; and bleeding and necrosis occurred in large areas. The three histological types of PTB present a progressively increasing degree of tumor malignancy. Potential malignancy may exist even in benign PTB. Therefore, PTB can be regarded as a general term of a series of fibrous epithelial tumors with different clinical courses and histopathological features.

Breast phyllodes tumors can occur at any age and is more common in women aged 35–55 years 3. Patients with PTB have a long clinical history of slow tumor growth and often complain of the recent rapid growth of a tumor mass. The tumor size of PTB widely varies, ranging from <1 cm to 40 cm 4. Surgical resection is the preferred treatment; however, the selection of the surgical approach depends on accurate preoperative diagnosis. Existing diagnostic methods for PTB have a low accuracy, irrespective of ultrasound, mammography, magnetic resonance imaging, or core needle biopsy 5, 6, 7. The uncertainty of preoperative diagnosis creates obstacles for developing reasonable surgical treatment regimens for PTB. Borderline and malignant PTBs have the prognostic feature of high local recurrence and also have a risk of distant metastasis 5, 8. The causes and risk factors for local recurrence of PTB remain inconclusive. A number of studies have reported that the selection of a surgical approach and the status of surgical margins are critical factors affecting postoperative tumor recurrence 9, 10, 11, 12, 13. The efficacy of postoperative adjuvant therapy is currently uncertain in PTB, and this type of tumor is generally insensitive to chemotherapy. Morales‐Vasquez et al. 14 detected no significant difference in the survival rate of patients undergoing postoperative adjuvant chemotherapy with doxorubicin and dacarbazine compared with those without chemotherapy. Reliable evidence is currently lacking for the efficacy of adjuvant radiotherapy. According to the database of the Surveillance, Epidemiology and Results Program (SEER), approximately 50% of patients with malignant PTB first received breast‐conserving surgery, whereas less than 5% of these patients received adjuvant radiotherapy after surgery 15. Moreover, Belkacemi et al. 16 and Barth et al. 17 suggested that adjuvant radiotherapy could reduce the risk of the postoperative recurrence of malignant PTB.

The control of local recurrence is a major problem that needs to be solved via timely clinical diagnosis and effective treatment for this type of tumor. However, clinical research on PTB has been limited, with a small sample size and a scarcity of comprehensive clinical analysis data. Particularly, studies concerning the certainty of efficacy of postoperative adjuvant therapy are rare, reflecting the low incidence of PTB, a small number of cases, difficult preoperative diagnosis and prediction of biological behavior, and limited radiotherapy techniques and equipment in most medical institutions. Reliable evidence is still lacking for the diagnosis and clinical treatment of PTB, while numerous clinical problems are pending. Therefore, we retrospectively collected and analyzed the clinicopathologic data of PTB patients from Fudan University Shanghai Cancer Center (FUSCC) in China. A detailed analysis was conducted on the postoperative recurrence and metastasis patterns of PTB and related risk factors. The clinical significance of the surgical approach selection and adjuvant radiotherapy was assessed with regard to the control of local recurrence of PTB.

## Patients and Methods

### Patients

The study included 454 PTB patients from 1 January 2002 to 1 April 2013. The inclusion criteria were (1) patients with primary or recurrent PTB who were admitted to FUSCC; if the initial surgery at other hospitals, the initial operation records, and postoperative pathologic examination results should be complete, and the pathological sections from other hospitals should be reviewed by pathologists at FUSCC to confirm the diagnosis, (2) a complete clinical medical history and pathological diagnosis data, especially the initial operation records, (3) the histological type of PTB in accordance with the standards for PTB developed by the WHO in 2003 1, and (4) personal information for the patients. The exclusion criteria were (1) patients who were admitted to FUSCC for the first time due to tumor recurrence but lacked consultation of pathological sections for surgical specimens of the primary tumor, (2) association with other types of malignant tumors, and (3) failure to contact due to change in contact number or address or rejection of any form of clinical follow‐up, which resulted in a loss to follow‐up. A total of 404 patients were finally included in the present study. The study was approved through the Ethics Committee of FUSCC. The patients were not required to sign an informed consent form in the retrospective study.

### Follow‐up and outcome

The follow‐up by the electronic medical record system included patients' baseline characteristics, preoperative examination, surgical records, and postoperative detailed pathological description and diagnosis reports. The follow‐up by phone or mail included important information on the medical history that was not acquired from the electronic medical record system, detailed process of visits to other hospital(s), especially the initial operation records and postoperative pathologic examination results, the frequency of postoperative reexamination and date and result of the last breast examination, tumor recurrence or metastasis or not, and tumor‐related death or not. Cases were lost to follow‐up if a large amount of clinical data was missing, or if the patient rejected follow‐up due to personal reasons, or if the contact failed. Recurrence‐free survival (RFS) was the primary outcome of this retrospective cohort study, defined as the survival time interval from postoperation to any recurrence, metastasis, death, or other types of failure resulting from PTB, whether the initial surgery at our hospital or other hospitals.

### Review of the surgical pathology sections

Two pathologists from FUSCC reviewed the surgical pathology sections. The review of pathological sections primarily included (1) histological type: benign, borderline, or malignant; (2) tumor residue: absent or present; (3) mitotic figures: mitotic figures were observed and counted in 10 continuous HPF at the most dense area of the cell, and the results were recorded as 0–3 mitotic figures/10 HPF, 4–9 mitotic figures/10 HPF, or ≥10 mitotic figures/10 HPF; (4) stromal cell hyperplasia: mainly refers to stromal overgrowth and hypercellularity, based on the area ratio of tumorous stromal components in 1 HPF, hyperplasia was recorded as mild (<1/3), moderate (1/3–2/3), and severe (>2/3); (5) stromal cell atypia: atypia was ranked into mild, moderate, or severe grades according to the size, shape difference, and color intensity of stromal cell nuclei in the tumor; (6) tumor border: clear or infiltrative; (7) tumor necrosis: present or absent; and (8) surgical margin: negative or positive 18.

### Statistical analysis

Survival analysis was conducted using the Kaplan–Meier method. The difference in recurrence‐free survival (RFS) between groups was determined using the log‐rank test. Multivariate analysis was performed using the Cox proportional hazards model, and the analysis only included significant variables (*P *<* *0.05) based on univariate Cox regression analysis or prognosis‐associated risk factors identified by previous reports to establish the model. All hypothesis tests were two‐sided. The difference was considered statistically significant at *P *<* *0.05. All these analyses were conducted using SPSS Statistics v22.0 (IBM SPSS, New York). A nomogram was generated based on the results of multivariate Cox analysis using the rms package in R version 3.3.1 (http://www.r-project.org/). The performance of the nomogram was measured using Harrell's concordance index (C‐index) and assessed by comparing nomogram‐predicted versus observed Kaplan–Meier estimates of survival probability. Bootstraps with 1000 resample were used for these activities. The accuracy of the prognostic prediction increased with increasing value of C‐index 19.

## Results

### Clinicopathologic features in different histological types

A total of 404 patients with PTB were included in this study. All patients were women, with a mean age of 41 years (range: 12–72 years) and a median follow‐up time of 46 months (range: 10–145 months). The distribution of clinicopathologic features in different histological types of the primary tumor is shown in Table 1.

**Table 1:** Clinicopathologic features of 404 phyllodes tumors of the breast‐based histological type (*N* = 404)

Clinicopathologic features	Histological type			*P* value
	Benign (%)	Borderline (%)	Malignant (%)	
Age				
<41	83 (49.4)	72 (39.1)	20 (38.5)	0.114
≥41	86 (50.6)	112 (60.9)	32 (61.5)	
Tumor size (mm)				
<50	129 (77.7)	115 (65.7)	21 (43.8)	<0.001
≥50	37 (22.3)	60 (34.3)	27 (56.3)	
Menopausal status				
Premenopausal	140 (84.3)	140 (76.9)	31 (59.6)	0.001
Postmenopausal	26 (15.7)	42 (23.1)	21 (40.4)	
Fibroadenoma surgery history				
No	136 (88.3)	140 (77.8)	33 (63.5)	<0.001
Yes	18 (11.7)	40 (22.2)	19 (36.5)	
Tumor rapid enlargement				
No	83 (50.6)	88 (49.7)	10 (20.4)	<0.001
Yes	81 (49.4)	89 (50.3)	39 (79.6)	
Tumor location				
Left breast	86 (51.2)	101 (54.9)	28 (53.8)	0.782
Right breast	82 (48.8)	83 (45.1)	24 (46.2)	
Surgery methods				
SE	158 (94.0)	150 (81.5)	35 (67.3)	<0.001
WLE	10 (6.0)	21 (11.4)	4 (7.7)	
Mastectomy	0 (0)	13 (7.1)	13 (25.0)	
Surgical margin				
Negative	150 (100)	156 (98.1)	41 (95.3)	0.060
Positive	0 (0)	3 (1.9)	2 (4.7)	
Tumor residual				
No	151 (99.3)	154 (99.4)	30 (93.8)	0.020
Yes	1 (0.7)	1 (0.6)	2 (6.3)	
Tumor margin				
Clear	150 (100)	152 (89.4)	20 (48.8)	<0.001
Infiltrative	0 (0)	18 (10.6)	21 (51.2)	
Mitosis per 10 HPF				
0–3	145 (100)	15 (8.5)	2 (4.1)	<0.001
4–9	0 (0)	151 (85.3)	5 (10.2)	
More than 10	0 (0)	11 (6.2)	42 (85.7)	
Stromal cell hyperplasia				
Low	147 (89.1)	92 (52.0)	9 (18.4)	<0.001
Moderate	18 (10.9)	82 (46.3)	17 (34.7)	
Severe	0 (0)	3 (1.7)	23 (46.9)	
Stromal cell atypia				
Low	156 (94.5)	33 (18.6)	4 (98.2)	<0.001
Moderate	9 (5.5)	143 (80.8)	26 (53.1)	
Severe	0 (0)	1 (0.6)	19 (38.8)	
Tumor necrosis				
No	162 (98.2)	158 (89.3)	26 (53.1)	<0.001
Yes	3 (1.8)	19 (10.7)	23 (46.9)	
Adjuvant RT				
No	168 (100)	179 (97.3)	47 (90.4)	<0.001
Yes	0 (0)	5 (2.7)	5 (9.6)	
Local recurrence				
No	162 (96.4)	158 (85.9)	30 (57.7)	<0.001
Yes	6 (3.6)	26 (14.1)	22 (42.3)	
Metastasis				
No	168 (100)	184 (100)	48 (92.3)	<0.001
Yes	0 (0)	0 (0)	4 (7.7)	
PTB caused death				
No	168 (100)	184 (100)	47 (90.4)	<0.001
Yes	0 (0)	0 (0)	5 (9.6)	

### Tumor recurrence and metastasis patterns

The 1‐, 3‐, 5‐, and 10‐year RFS rates of all 404 patients were 94.8%, 88.4%, 87.6%, and 86.6%, respectively. The 1‐, 3‐, 5‐, and 10‐year RFS rates of 168 benign PTB patients were 99.4%, 97.0%, 97.0%, and 96.4%, respectively. The 1‐, 3‐, 5‐, and 10‐year RFS rates of 184 borderline PTB patients were 97.3%, 88.0%, 86.4%, and 85.9%, respectively. The 1‐, 3‐, 5‐, and 10‐year RFS rates of 52 malignant PTB patients were 71.2%, 61.5%, 59.6%, and 57.7%, respectively. Significant differences in the 1‐, 3‐, 5‐, and 10‐year RFS rates of benign, borderline, and malignant PTBs were observed (*P *<* *0.05). The RFS rate of PTB significantly decreased with increasing histological grade of the tumor.

A total of 54 PTB patients had postoperative local recurrence, with an overall local recurrence rate of 13.7%. The recurrent patients included six benign cases, 26 borderline cases, and 22 malignant cases. The local recurrence rates of benign, borderline, and malignant PTBs were 3.6%, 14.1%, and 42.3%, respectively. With regard to surgical approach, 48 (88.9%) of the 54 recurrence patients received simple excision (SE), and only two patients (3.7%) received wide local excision (WLE); the remaining four patients (7.4%) received mastectomy. Fifty cases (92.6%) recurred in the ipsilateral breast, while four cases (7.4%) recurred in the ipsilateral chest wall after mastectomy. Six patients (11.1%) received adjuvant radiotherapy, and 48 patients (88.9%) did not. Three recurrent patients had tumor metastasis, and three patients died as a result of tumor progression. Regarding histological features, one patient had a positive surgical margin, two patients had tumor residue, 18 patients had an infiltrative tumor border, and 11 patients had tumor necrosis. Most recurrent patients had ≥10 mitotic figures/10 HPF, including 24 cases (44.4%). Stromal cell hyperplasia was mild, moderate, and severe in 18 (33.3%), 21 (38.9%), and 14 (25.9%) cases, respectively. Stromal cell atypia was mild, moderate, and severe in 9 (16.7%), 35 (64.8%), and 9 (16.7%) cases, respectively.

### Surgical treatment

One hundred and sixty‐eight patients with benign PTB were classified as the low‐risk group, all of which underwent surgical treatment through breast‐conserving surgery, including 158 SE patients (94.0%) and 10 WLE patients (6.0%). One hundred and eighty‐four patients with borderline PTB and 52 patients with malignant PTB were classified as the high‐risk group, including 185 patients (78.4%) undergoing SE, 25 patients (10.6%) undergoing WLE, and 26 patients (11.0%) undergoing mastectomy.

Among 168 benign PTB patients, two (1.2%) patients underwent supplementary WLE of the primary tumor after SE and neither recurred after the second surgery.

Among 184 patients with borderline PTB, 43 (23.4%) patients underwent a second surgery of the primary tumor after SE, 40 patients underwent WLE as a complementary surgical approach after SE, and two cases recurred; three patients underwent mastectomy as a supplementary surgery after SE and none recurred.

Among 52 patients with malignant PTB, 15 (28.8%) patients underwent a second surgery for the primary tumor. Among these patients, one of four patients (25.0%) undergoing supplementary WLE after SE had tumor recurrence, whereas none of the 11 patients undergoing mastectomy after SE or WLE had tumor recurrence.

Among 54 locally recurrent patients, one malignant PTB patient was pathologically diagnosed for the first recurrence and underwent no surgical treatment. One of 14 malignant PTB patients with twice LR underwent no surgical treatment. At the first recurrence, most patients with benign PTB received SE treatment and only one underwent mastectomy, whereas seven patients with borderline PTB and 10 patients with malignant PTB underwent mastectomy. None of the patients with recurrent cases of benign PTB had a second recurrence. One patient with borderline PTB and three patients with malignant PTB who had a second recurrence underwent mastectomy. One patient with malignant PTB who had a third recurrence underwent mastectomy after the second recurrence; the third recurrence was observed in the ipsilateral chest wall, and the patient received radiotherapy to the chest wall (50 Gy/25 Fx) after SE.

### Adjuvant radiotherapy

Ten (2.5%) of the 404 patients received postoperative adjuvant radiotherapy. All 10 cases were of high‐risk patients, including five patients with borderline PTB and five patients with malignant PTB. In the radiotherapy group, 44.4% had an infiltrative tumor border, which was higher than that in the nonradiotherapy group (17.3%) (*P *=* *0.040). In the radiotherapy group, the majority of patients had severe stromal cell hyperplasia. In the nonradiotherapy group, most patients had mild stromal cell hyperplasia. The difference between the two groups was statistically significant (*P *<* *0.001). Both local recurrence and distant metastasis were higher in the radiotherapy group compared with the nonradiotherapy group (*P *<* *0.05). Among 10 patients receiving adjuvant radiotherapy, only four underwent radiotherapy after primary tumor surgery. Specifically, two patients received radiotherapy to the whole breast (50 Gy/25 Fx) after breast‐conserving surgery, and two patients received radiotherapy to the chest wall (50 Gy/25 Fx) after mastectomy. There was no tumor recurrence or metastasis after radiotherapy. One patient with borderline PTB underwent WLE after the second recurrence; this patient received radiotherapy to the whole breast (50 Gy/25 Fx) after surgery, with a tumor bed boost of 10 Gy/5 Fx. Five patients received radiotherapy after surgery at the first recurrence; according to the surgical approach for recurrent lesions, one patient received radiotherapy to the whole breast (50 Gy/25 Fx) after breast‐conserving surgery, with a tumor bed boost of 10 Gy/5 Fx, whereas four patients received radiotherapy to the chest wall (50 Gy/25 Fx) after mastectomy. None of the 10 patients experienced recurrence after receiving adjuvant radiotherapy. One patient with malignant PTB underwent adjuvant radiotherapy after surgery at recurrence; tumor metastasis and death occurred in this case after radiotherapy to the chest wall (50 Gy/25 Fx).

### Univariate and multivariate analysis

A total of 404 patients were divided into low‐risk and high‐risk groups based on the histological type of PTB. The low‐risk group included 168 patients with benign PTB, and the high‐risk group included 236 patients with borderline or malignant PTB.

**Figure 1:**
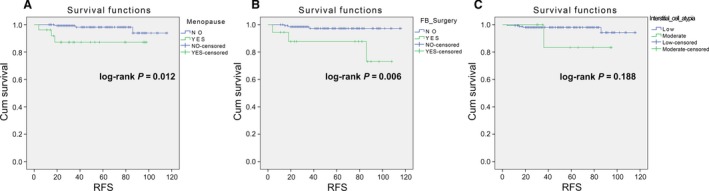
Log‐rank test for low‐risk PTB. (A) Premenopausal versus Postmenopausal (log‐rank *P *=* *0.012); (B) With a history of fibroadenoma surgery versus without a history of fibroadenoma surgery (log‐rank *P *=* *0.006); (C) Interstitial (stromal) cell atypia (low vs. moderate, log‐rank *P *=* *0.188).

The low‐risk group (*N *=* *168) showed that postmenopausal onset and a history of fibroadenoma surgery were risk factors for postoperative RFS in univariate analysis (Table 2, Fig. 1). The recurrence risk of the postmenopausal onset group was higher than that in the premenopausal onset group (Hazard ratio = 6.10, 95% CI: 1.22–30.37, *P *=* *0.027). The recurrence risk of the group with a history of fibroadenoma surgery was higher than the group without a history of fibroadenoma surgery (Hazard ratio = 7.29, 95% CI: 1.43–37.20, *P *=* *0.017). In other word, PTB patients, with the history of breast fibroadenoma excision, are more likely to relapse. However, multivariate Cox regression analysis revealed that there was no independent prognostic factor for RFS (Table 2).

**Table 2:** Univariate and multivariate analysis for low‐risk PTB (*N* = 168)

Clinicopathologic features	*N*	LR (%)	Crude HR (95% CI)	Adjust HR (95% CI)	*P* value (Cox regression)	
					Univariate	Multivariate
Age (year)						
<38	72	1 (1.4)	–		0.221	–
≥38	96	5 (5.2)	3.82 (0.45–32.74)			
Tumor size (mm)						
<50	129	4 (3.1)	–		0.823	–
≥50	37	1 (2.7)	0.78 (0.09–7.00)			
Menopausal status						
No	140	3 (2.1)	–	–	0.027	0.315
Yes	26	3 (11.5)	6.10 (1.22–30.37)	2.668 (0.393–18.098)		
Fibroadenoma surgery						
No	136	3 (2.2)	–	–	0.017	0.180
Yes	18	3 (16.7)	7.29 (1.43–37.20)	3.632 (0.552–23.912)		
Tumor rapid enlargement						
No	83	1 (1.2)	–		0.152	–
Yes	81	5 (6.2)	4.81 (0.56–41.22)			
Tumor location						
Left breast	86	3 (3.5)	–		0.923	–
Right breast	82	3 (3.7)	1.08 (0.22–5.37)			
Surgery methods						
SE	158	6 (3.8)	–		0.652	–
WLE	10	0 (0)	–			
Tumor residual						
No	151	5 (3.3)	–		0.905	–
Yes	1	0 (0)	–			
Stromal cell hyperplasia						
Low	147	5 (3.4)	–		0.586	–
Moderate	18	0 (0)	–			
Stromal cell atypia						
Low	156	4 (2.6)	–	–	0.222	0.281
Moderate	9	1 (11.1)	3.92 (0.44–35.13)	3.630 (0.349–37.803)		
Tumor necrosis						
No	162	5 (3.1)	–		0.853	–
Yes	3	0 (0)	–			

**Figure 2:**
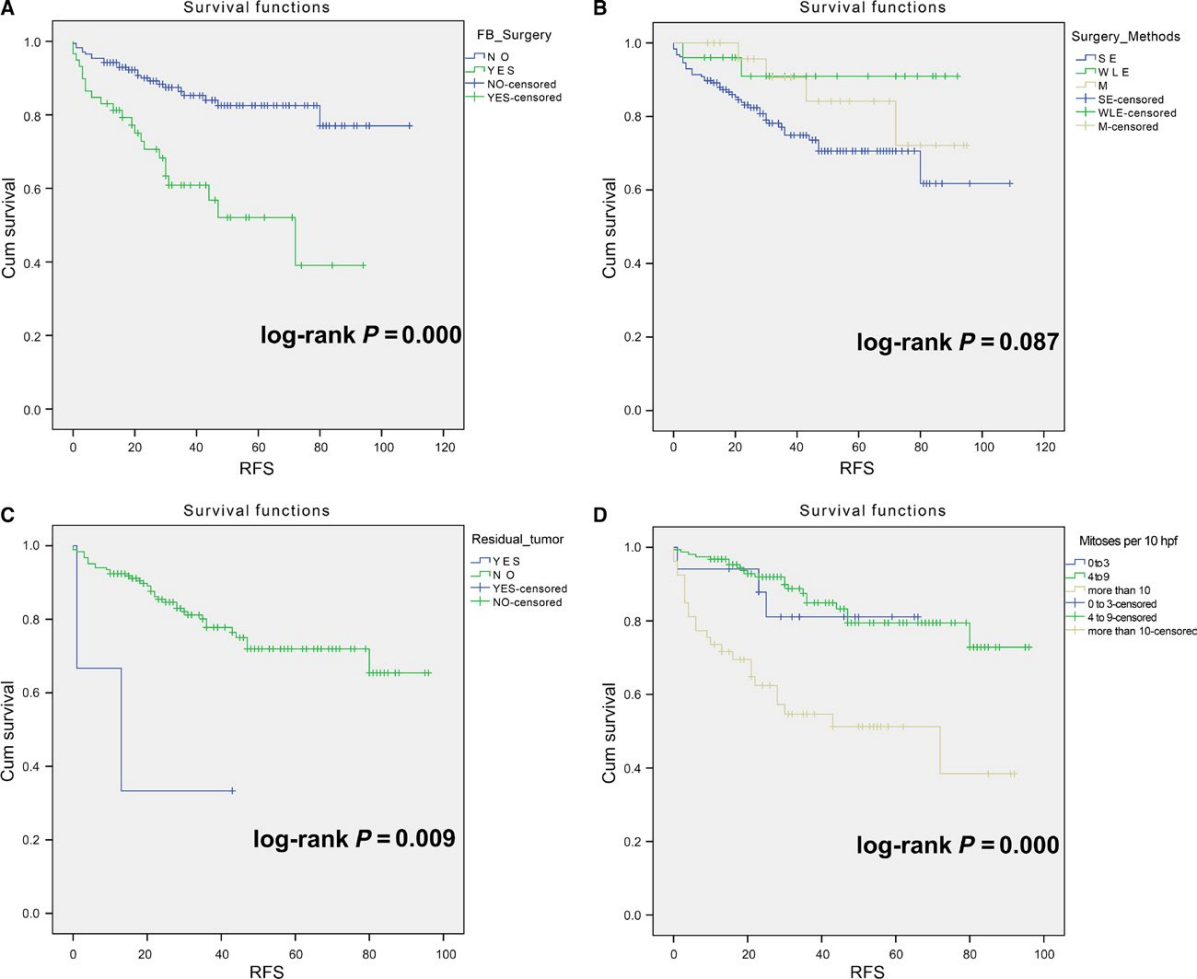
Log‐rank test for high‐risk PTB. (A) With fibroadenoma surgery history versus without a history of fibroadenoma surgery (log‐rank *P *<* *0.0001); (B) Surgery methods (SE vs. WLE vs. M, log‐rank *P *=* *0.087); (C) With tumor residual versus without tumor residual (log‐rank *P *=* *0.009); (D) Mitosis per 10 HPF (0–3 vs. 4–9 vs. more than 10, log‐rank *P *<* *0.0001). M, mastectomy.

**Figure 3:**
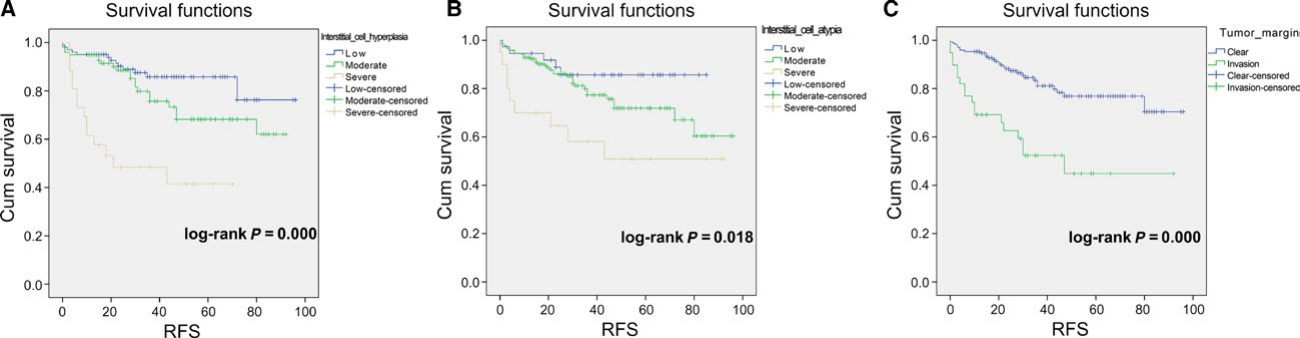
Log‐rank test for high‐risk PTB. (A) Interstitial (stromal) cell hyperplasia (low vs. moderate vs. high, log‐rank *P *<* *0.0001); (B) Interstitial (stromal) cell atypia (low vs. moderate vs. high, log‐rank *P *=* *0.018); (C) Tumor border (clear vs. invasion, log‐rank *P *<* *0.0001).

**Figure 4:**
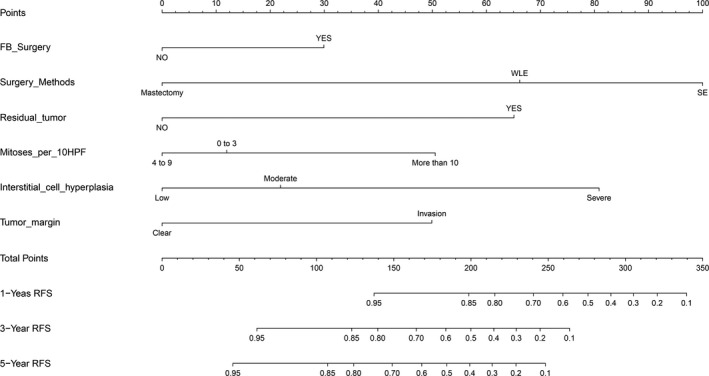
Nomogram for predicting recurrence‐free survival (RFS) of patients with phyllodes tumors. To use the nomogram, locate the first variable. Draw a line straight upwards to the Points axis to determine the number of points received for the variable. Repeat this process for the other variables, and sum up the points achieved for each variable. The sum of these numbers is located on the Total Points axis, and a line is drawn downwards to the survival axes to determine the likelihood of 1‐, 3‐, and 5‐year RFS.

**Figure 5:**
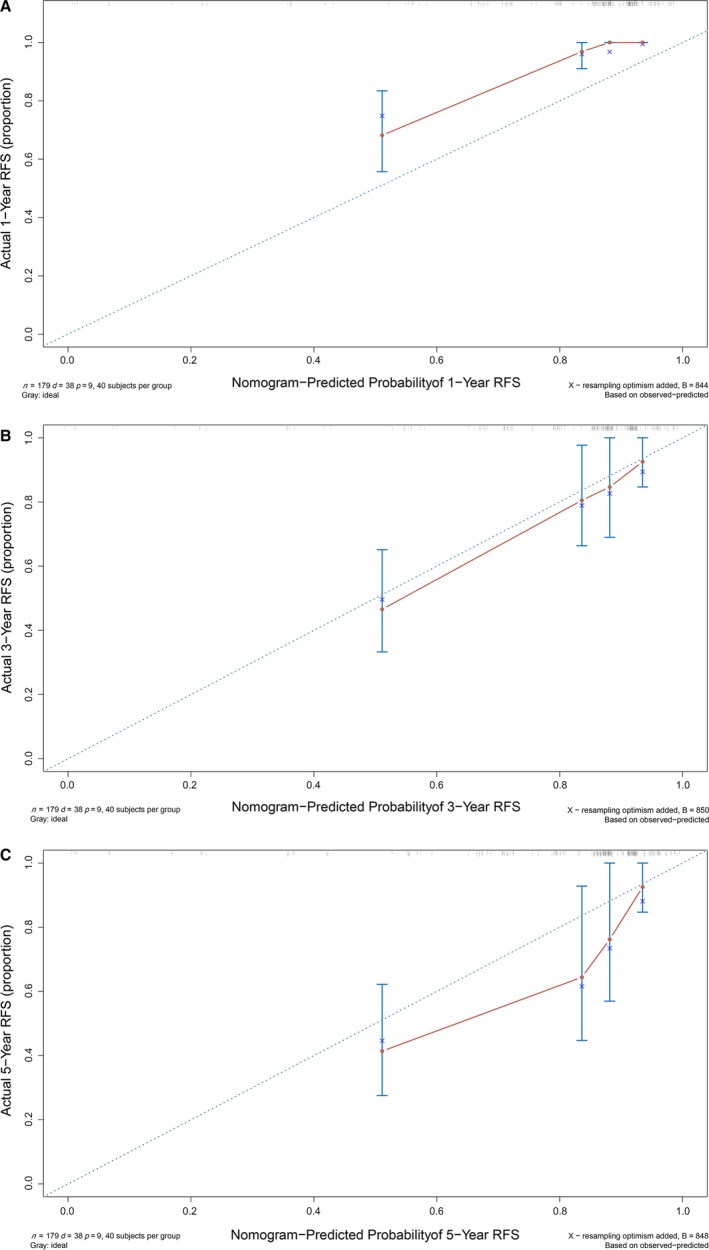
Bootstrapped estimates of calibration accuracy at (A) 1‐year RFS, (B) 3‐year RFS, and (C) 5‐year RFS. The ideal outcome (dot line) and the observed outcome (maroon line) are depicted. This figure demonstrates how accurately predictions at different risk levels conform to observed outcomes for the nomogram.

The high‐risk group (*N *=* *236) showed that history of fibroadenoma surgery, tumor residue, infiltrative tumor border, stromal cell hyperplasia, stromal cell atypia, and mitotic activity were high‐risk prognostic factors for RFS in univariate analysis (Table 3, Figs 2 and 3). Multivariate Cox regression analysis revealed that surgical approach (*P *=* *0.015) and tumor margin (*P *=* *0.014) were two independent prognostic factors for RFS (Table 3). The nomogram, generated based on multivariate Cox regression coefficients, is shown in Figure 4. Figure 5 demonstrates the bootstrap estimates of calibration accuracy for 1‐, 3‐, and 5‐year RFS estimates from the final Cox model. The nomogram with a higher C‐index predicted RFS (C‐index = 0.835, SE = 0.050).

**Table 3:** Univariate and multivariate analysis for high‐risk PTB (*N* = 236)

Clinicopathologic features	*N*	LR (%)	Crude HR (95% CI)	Adjust HR (95% CI)	*P* value (Cox regression)	
					Univariate	Multivariate
Age (year)						
<38	99	25 (25.3)	–		0.091	–
≥38	137	23 (16.8)	0.61 (0.35–1.08)			
Tumor size (mm)						
<50	136	23 (16.9)	–		0.403	–
≥50	87	13 (14.9)	0.75 (0.38–1.48)			
Menopausal status						
Premenopausal	171	39 (22.8)	–		0.152	–
Postmenopausal	63	9 (14.3)	0.59 (0.29–1.21)			
Fibroadenoma surgery history						
No	173	24 (13.9)	–	–	<0.0001	0.110
Yes	59	23 (39.0)	3.37 (1.90–5.98)	1.772 (0.878–3.575)		
Tumor rapid enlargement						
No	98	17 (17.3)	–		0.587	–
Yes	128	28 (21.9)	1.18 (0.65–2.17)			
Tumor location						
Left breast	129	28 (21.7)	–		0.598	–
Right breast	107	20 (18.7)	0.86 (0.48–1.52)			
Surgery methods						
SE	185	42 (22.7)	–	–	0.110	0.015
WLE	25	2 (8.0)	0.28 (0.07–1.18)	0.488 (0.109–2.181)		
M	26	4 (15.4)	0.50 (0.18–1.41)	0.147 (0.038–0.566)		
Surgical margin						
Negative	197	45 (22.8)	–		0.776	–
Positive	5	1 (20.0)	0.75 (0.10–5.45)			
Tumor residual						
No	184	37 (20.1)	–	–	0.020	0.117
Yes	3	2 (66.7)	5.45 (1.30–22.76)	4.077 (0.704–23.618)		
Tumor margin						
Clear	172	29 (16.9)	–	–	<0.0001	0.014
Infiltrative	39	18 (46.2)	3.45 (1.91–6.23)	2.731 (1.229–6.068)		
Mitosis per 10 HPF						
0–3	17	3 (17.6)	–	–	<0.0001	0.139
4–9	156	21 (13.5)	0.79 (0.23–2.65)	0.994 (0.229–4.310)		
More than 10	53	24 (45.3)	3.20 (0.96–10.66)	2.517 (0.515–12.316)		
Stromal cell hyperplasia						
Low	101	13 (12.9)	–	–	<0.0001	0.090
Moderate	99	21 (21.2)	1.73 (0.87–3.47)	1.480 (0.655–3.347)		
Severe	26	14 (53.8)	5.96 (2.79–12.74)	3.961 (1.150–13.640)		
Stromal cell atypia						
Low	37	5 (13.5)	–	–	0.026	0.724
Moderate	169	34 (20.1)	1.82 (0.71–4.65)	0.677 (0.213–2.149)		
Severe	20	9 (45.0)	4.11 (1.37–12.30)	0.904 (0.190–4.299)		
Tumor necrosis						
No	184	37 (20.1)	–		0.403	–
Yes	42	11 (26.2)	1.33 (0.68–2.62)			

## Discussion

In the present study, surgical approach and tumor border were revealed as independent prognostic factors for RFS in the high‐risk group (borderline and malignant PTBs), and patients who received simple excision with an infiltrative tumor border had a higher recurrence rate. In the low‐risk group (benign PTBs), only six patients experienced recurrence; thus, in the multivariate analysis, there were no positive findings. The histological type of PTB was closely associated with postoperative tumor recurrence; the higher the histological grade, the higher the recurrence rate (*P *<* *0.001). All metastatic cases were malignant, and the distant metastasis rate was 1.0% (4/404). Most studies showed that the recurrence rate is lower for benign PTB and highest for malignant PTB.

However, simple histological grading cannot effectively guide clinical treatment and prognosis; a comprehensive consideration of the role of histopathological indices is of greater guiding value to determine prognosis 20, 21, 22. Taira et al. 23, Asoglu et al. 24, and Chaney et al. 25 proposed that stromal cell atypia is the only independent pathological prognostic factor for local tumor recurrence. Sawalhi et al. 26 affirmed that patients with moderate or severe stromal cell atypia have a poor prognosis. Gnerlich et al. 27 showed that stromal cell hyperplasia and tumor necrosis are prognostic factors for benign PTB and borderline and malignant PTBs. Tan et al. 20 reported that the malignant degree of stromal cells is an independent prognostic factor for tumor RFS; furthermore, mitotic activity, stromal cell hyperplasia, stromal cell atypia, and tumor border were included in the nomogram risk prediction model, which could accurately predict the 1‐, 3‐, 5, and 10‐year RFS in individual patients. Belkacemi et al. 16 showed that tumor residue is a risk factor for postoperative recurrence. Roa et al. 28, Barrio et al. 21, and Ben et al. 12 showed that a higher mitotic count predicts a worse prognosis.

Existing studies support that the selection of the surgical approach for PTB and the status of the surgical margin are influencing factors of postoperative recurrence 22, 26, 27, 29. Sotheran et al. 9 and Haberer et al. 10 highlighted the importance of WLE of breast tumors to control postoperative recurrence in borderline and malignant PTBs. Bhargav et al. 11 proposed that irrespective of the histological grade, WLE is the preferred surgical approach, while mastectomy is required for all recurrent cases. Ben Hassouna et al. 12 proposed that mastectomy is the preferred surgical approach for malignant PTB. However, Kapiris et al. 13 detected no significant difference in patients with malignant PTB who underwent WLE and mastectomy; these authors proposed that a negative surgical margin is the prerequisite to control the recurrence and distant metastasis of malignant PTB. Pandey et al. 30 showed that surgical margin is an independent risk factor for recurrence of PTB, which improves disease‐free survival and reduces the likelihood of local recurrence. Mangi et al. 31 showed that all recurrent cases had a surgical margin <1 cm. However, Lenhard 32 and Cheng et al. 33 studied surgical margin and observed no difference between the recurrence group and nonrecurrence group. Additionally, Jang et al. 29 observed no advantage of positive surgical margins >1 cm compared with those smaller than 1 cm. Fou et al. 34 showed that a higher long‐term survival rate could be achieved in malignant PTB by local excision to ensure a negative surgical margin. In the current study, univariate analysis revealed that the selection of the surgical approach was not significantly associated with tumor recurrence in the low‐risk or high‐risk group (*P *=* *0.652). Multivariate analysis revealed that the selection of the surgical approach was an independent prognostic factor for postoperative RFS in high‐risk patients (*P *=* *0.015). Because positive surgical margin was detected in only six patients, we could not demonstrate the effect of surgical margin on tumor RFS.

The efficacy of adjuvant radiotherapy for PTB remains uncertain. A relatively consistent conclusion from existing studies is that adjuvant radiotherapy can reduce the recurrence rate of PTB with a higher degree of malignancy 16. Pezner et al. 35 noted the significantly improved value of radiotherapy as an adjuvant therapy in cases with a tumor size >2 cm undergoing local excision and those with a tumor size >10 cm undergoing mastectomy. Pandey et al. 30 observed a higher 5‐year disease‐free survival rate in malignant tumor patients who underwent adjuvant radiotherapy compared with that in patients in the nonradiotherapy group (61% vs. 25%); however, the difference between the two groups was not statistically significant (*P *=* *0.16). Belkacemi et al. 16 reported that postoperative adjuvant radiotherapy improved the 10‐year local control rate of PTB in borderline and malignant groups without affecting overall survival. Gnerlich et al. 27 showed that postoperative adjuvant radiotherapy significantly reduced the local recurrence rate without benefiting disease‐free survival and overall survival. Barth et al. 17 showed that adjuvant radiotherapy was an effective treatment for the control of postoperative recurrence of borderline and malignant PTBs with a negative surgical margin following breast‐conserving surgery; the recurrence rate was markedly reduced in patients with a negative surgical margin after breast‐conserving surgery who underwent adjuvant radiotherapy compared with that in the nonradiotherapy group. In the present study, there was no local recurrence in any of the 10 patients who received adjuvant radiotherapy, while both the local recurrence rate and distant metastasis rate were higher compared with those of the nonradiotherapy group (*P *<* *0.05). On the one hand, the radiotherapy patients were primarily those who had undergone postoperative recurrence and a second resection of a recurrent lesion, with clinical selectivity. On the other hand, the sample size was small, resulting in a lack of reliability in the comparative analysis of the data.

In conclusion, the present study demonstrated that surgical approach and tumor border are independent prognostic factors for RFS in borderline and malignant PTBs. Thus, it is recommended that the need and approach of clinical management based on comprehensive consideration of the histological type, tumor border, tumor residue, mitotic activity, and degree of stromal cell hyperplasia and atypia should be considered. This predictive nomogram based on clinicopathologic features and surgical approaches can be applied for patient counseling and the clinical management of PTB. The efficacy of adjuvant radiotherapy remains uncertain.

## Conflict of Interest

The authors declare no conflict of interests.
